# A Study Protocol for the Management of Children With Juvenile Idiopathic Arthritis Based on ePROs

**DOI:** 10.3389/fped.2022.905182

**Published:** 2022-07-06

**Authors:** Biyu Shen, Songsong Shi, Hengmei Cui, Yunyun Li, Haoyang Chen, Huan Jin, Jia Xu, Zuojia Liu, Yanliang Jin

**Affiliations:** ^1^Department of Nursing, Shanghai Children's Medical Center, Shanghai, China; ^2^School of Nursing, Shanghai Jiao Tong University, Shanghai, China; ^3^Department of Rheumatology, Shanghai Children's Medical Center, Shanghai, China

**Keywords:** juvenile idiopathic arthritis, electronic patient-reported outcomes, symptom management, pediatric, PROMIS

## Abstract

**Background:**

Juvenile idiopathic arthritis (JIA) is a common chronic rheumatic disease with no known cures, affecting children with the age of onset under 16 years. Patient-reported outcome (PRO) measures are an important basis for evaluating the impact of JIA and associated therapies, however, which is particular challenge in the pediatric setting. At present, no randomized controlled studies have investigated the effect and usability of ePROs symptom management for children with JIA.

**Methods:**

This longitudinal, randomized, controlled trial will be carried out at outpatient and pediatric wards of the Shanghai Children's Medical Center affiliated to Shanghai Jiao Tong University School of Medicine. A total of one hundred children with JIA diagnosed according to the International League of Associations for Rheumatology (ILAR) patients are randomized to receive individualized symptom management based on ePROs or routine management. The primary outcome is the mean C-Ped-PROMIS T-scores of patients in the ePROs-based group and the control group. The secondary outcomes are the trajectories of C-Ped-PROMIS T-scores and HRQOL scores, and changing relationship between them. Data were collected at 5 time points: at enrollment (“baseline”) and at the time of follow-up visits scheduled at 1, 3, 6, and 12 months.

**Discussion:**

The findings are expected to conclude that the symptom management based on ePROs for children with JIA can improve the symptom of JIA, and it is a feasible and effective way to monitor and intervene children with JIA.

**Clinical Trial:**

http://www.chictr.org.cn/showproj.aspx?proj=132769; (ChiCTR2100050503)

## Introduction

Juvenile idiopathic arthritis (JIA), defined as arthritis of unknown cause with an onset before the age of 16 and persisting for at least 6 weeks (1), is the most common rheumatic condition in childhood (2). JIA is characterized by chronic synovial inflammation of joints (1), and its pathogenesis has not been fully elucidated. The annual incidence rate of juvenile arthritis (JA), including JIA, juvenile rheumatoid arthritis (JRA), and juvenile chronic arthritis (JCA), is 0.83–25 per 100 000, and the prevalence in China is reported to be 3.8 per 100 000. JIA involves multiple organs and in combination with pain, fatigue, and depression (3), most children with JIA need long-term pharmacological treatment and regular periodic clinical follow-ups (4), and some serious cases even need to require multiple hospitalizations. The primary symptoms of JIA are joint pain, stiffness, and lameness (5), which are associated with daily activities limitation, long-term disability, and reduced health-related quality of life (HRQOL) (6). These effects can persist into adult life, profoundly affecting generic health status and HRQOL (7).

Compared with healthy peers, the HRQOL of children with JIA is hindered seriously (8), and some of them are even much lower than those of other patients with chronic diseases. JIA can also impair the quality of life of their parents and families (9), with them reporting significant labor-time loss and healthcare costs (10).

Consequently, there is a growing consensus among researchers and clinicians that ameliorating patient symptoms and improving HRQOL are the primary goals of the management of JIA (9, 11). The constant monitoring of the JIA course (such as disease manifestations, therapies effects, morbidities, functional status, and quality of life) is a key way to achieve the goals. In the past few years, many feasible instruments specific to JIA have been developed for outcome measurement. For example, the core sets of disease activity measures are used to assess whether children with JIA show clinically improvement after anti-rheumatic drug treatment (12). Recently, Juvenile Arthritis Disease Activity Score (JADAS) (13), containing four measures, was developed to assess disease activity and disease damage. In addition, the patient/parent-acceptable symptom state was estimated by patient/parent acceptable symptomatic state (PASS) (14). However, these measures are not widely used in most pediatric centers. Many authorities now have focused on patient-reported outcomes (PROs), which provide an important way to evaluate the impact of JIA course and make more individualized symptom management and treatment, which is also an effective way to achieve patient-centered care.

The key feature of PROs (15) is patients provide health information directly. Common PROs include HRQL, health status, patient satisfaction and treatment experience, psychological distress, pain, and self-efficacy (16), and PROs have been widely applicated, particularly in the oncology domain (17). To deepen and promote the PROs idea, the United States National Institutes of Health (NIH), as the main sponsor, has been developing Patient Reported Outcomes Measurement Information System (PROMIS) since 2004 (18). Pediatric PROMIS (19), a pediatric self-report item banks, was created by University North Carolina (UNC), which has been validated to be sensitive to evaluate the PROs in children with chronic pain (20), children with cerebral palsy (21), children with Crohn's disease (22), and children with JIA (23). Pediatric PROMIS can also be used as clinical trial endpoints (24).

The research team of Chinese professor Changrong Yuan from Second Military Medical University has translated it into Chinese version of the Pediatric PROMIS (C-Ped-PROMIS) (25). The C-Ped-PROMIS contains eight short forms: depressive symptoms, anger, anxiety, fatigue, pain interference, peer relationships, mobility, and upper extremity function, which has been found to have good reliability and validity (25), and is suitable as a standardized tool for evaluating the PROs of children in our country. For 5- to 7-year-old pediatric child, they are too young to complete the PROs equipment. Thus, the PROMIS Parent Proxy Report Scales for Children is used to solve this problem, which has been studied as suitable for parents of children with the ages 5–7 (26). The PROMIS Parent Proxy Report Scales also have been authorized to translate to the Chinese version (25). For easy description, we refer to both Pediatric PROMIS and PROMIS Parent Proxy Report Scales collectively as C-Ped-PROMIS.

The PROs are originally paper-based through written questionnaires and/or in-person interviews (27), called “paper-based PROs.” With growing information technology, PROs are collected by devices or software, called “ePROs” (28), which were found to be more flexible, convenient, and efficient (29). Compared with paper-based PROs, the ePROs not only can avoid data entry errors and reduce missing information (30) but also can increase the overall survival among patients with cancer (31). The research team of professor Yuan has developed smartphone applications (apps) with “C-Ped-PROMIS” module to collect pediatric PROs (19). This app includes three modules: “Questionnaire,” “Mine,” and “Manor,” and it can implement multiple functionalities including collecting basic information about children and their parents, visualization of PROs, and a virtual animal adoption incentive system. The main function of the “Questionnaire” module is to collect basic information and the symptom assessment questionnaires, which includes three parts: basic information, parents' entrance, and children's entrance. Parents fill in basic information about themselves and their children, as well as childhood disease information. In the parents' entrance, the parents of children aged 5–7 complete the PROMIS Parent Proxy Report Scales. In the children's entrance, children aged eight, and older independently complete Pediatric PROMIS. Moreover, taking into account the specificity of children, the ePROs use the cartoon interface and equip with voice assistants, which can announce the content of each item. For the incentive system, it was designed that at the beginning of filling in the symptom questionnaire, there will be six virtual animals on the interface, and children can choose one of them. The virtual animals will grow as the children fill out the questionnaire carefully. In the “Manor,” children can see the growth status of the virtual animals. After completing the questionnaire for the last time, we will exchange an identical animal toy for children. Based on the app, we design a new ePROs app to evaluate, monitor, and manage the PROs of children with JIA. After authorization, we add the Pediatric Quality of Life Inventory^TM^3.0-Rheumatology Module (PedsQL^TM^3.0-RM) (32) to the “Questionnaire” module for assessing the HRQOL of patients. In addition, we incorporate in the app a “Symptom Intervention” module for performing targeted and individualized interventions of children with JIA dynamically. The ePROs allow children with JIA to self-report their individualized symptoms, and the arising real-time data are available to health professionals to provide symptom management.

In summary, the JIA diagnosis and treatment are a long-term, dynamic, and complex process. The timely and dynamic symptom intervention for children with JIA is necessary for the symptom experience, and treatment needs and therapeutic effects are varied. The present human randomized, controlled, longitudinal trial is designed to evaluate the effect of symptom management for children with JIA based on ePROs.

## Hypotheses And Objectives

### Hypotheses

The symptom management based on ePROs for children with JIA will improve the HRQOL of JIA, monitor, and intervene in the symptom of children with JIA feasibly and effectively.

### Objectives

The primary objective was to assess the effect of symptom management for patients with JIA based on ePROs by comparing the C-Ped-PROMIS T-scores of patients between the ePROs-based group and the control group.

The secondary objective of this trial was to assess what extent can symptom management based on ePROs improve the HRQOL of patients by evaluating the relationships between changes in C-Ped-PROMIS and changes in the HRQOL of patients.

## Methods And Analyses

### Design and Setting

**Figure 1:**
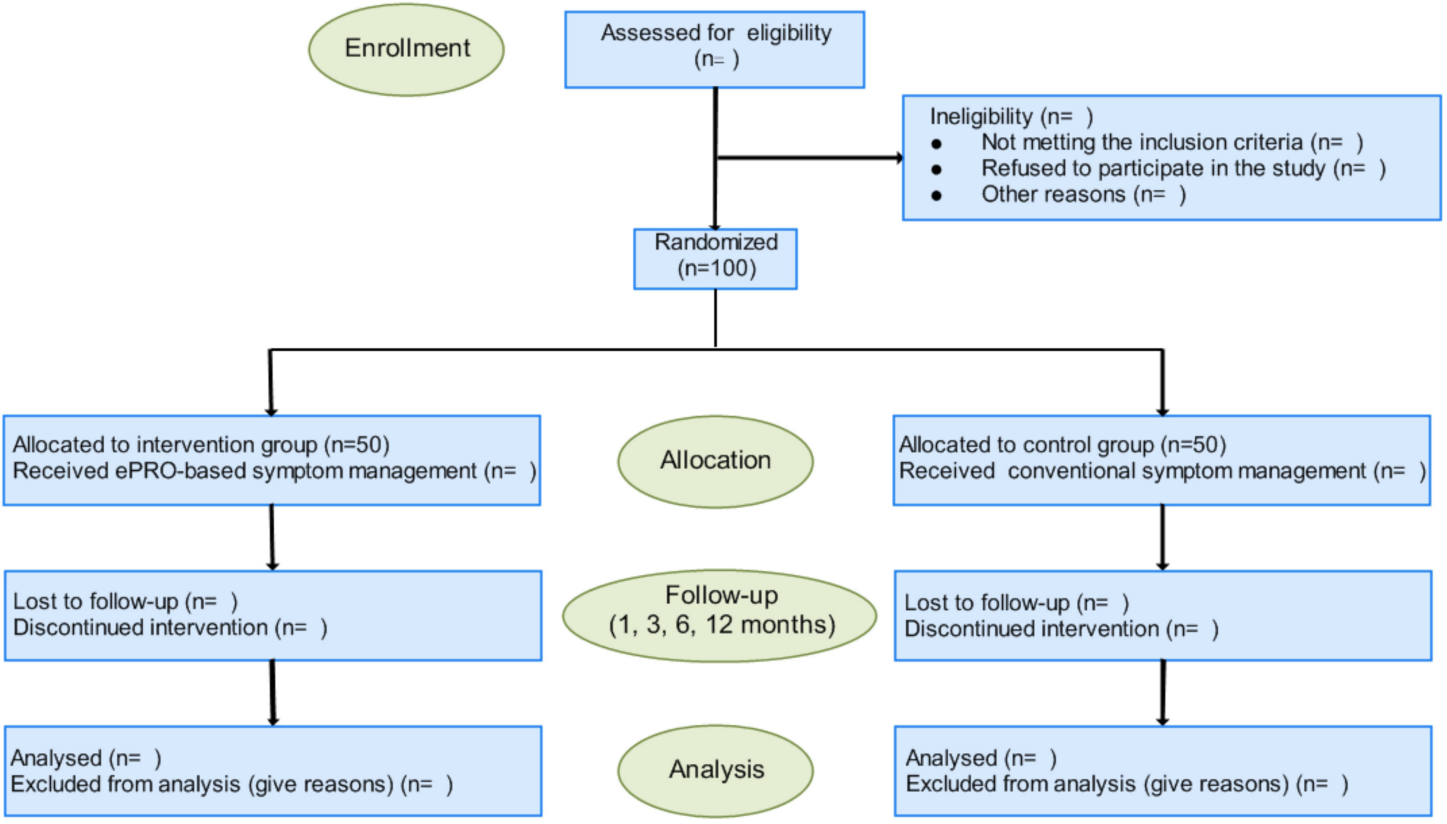
Flow diagram of the trial.

A longitudinal, randomized controlled trial will be carried out in outpatient and pediatric wards of the Shanghai Children's Medical Center (SCMC) affiliated to Shanghai Jiao Tong University School of Medicine between 1 September 2021 and 31 August 2023. The SCMC, a children' specialized hospital (level 3), is one of the main units of the National Children's Medical Center (NCMC). The number of patients with JIA in SCMC is approximately 1,500 per year. The implementation and reporting of this protocol will adhere to the standard protocol items: Recommendations for Interventional Trials (33) and the guidelines of Consolidated Standards of Reporting Trials (34). The trail is illustrated in a flowchart as shown in Figure 1.

### Participants

Participants are recruited from the outpatient and inpatient clinics. The main inclusion criteria for this trial are as follows: (1) meeting the diagnostic criteria for JIA of the Pediatric Rheumatology International Trials Organization (PRINTO); (2) aged 5 to 17 years; (3) the course of the disease is 3 months or more, and the family members have a certain degree of understanding of the disease; (4) patients and parents have normal understanding and expression skills and able and agree to respond to the questionnaire on the WeChat mini-program. Children diagnosed with early-onset ANA-positive JIA, other JIA, and unclassified JIA, or who combined with other major diseases that affect the quality of life, such as severe infections, are excluded.

### Consent

Following recruitment, a consent form is sent to the patient and family (parent/guardian) participants. The investigators explain the purpose and process of this study to the patient and family (parents/guardian), and they can put any questions to the investigators. Patients and their families are free to withdraw consent at any time during the study period (including the intervention period). Finally, they are required to sign the consent form.

### Sample Size Calculation

Sample size was calculated using the primary outcome –Pediatric Patient Reporting outcomes (Profile 25), and the “two mean comparison formula” as follows (35, 36): $n_{2}=\frac{{\left(z_{1-\alpha /2}+z_{1-\beta }\right)}^{2}(sd_{1}{}^{2}+sd_{2}{}^{2})(1+1/k)}{2{\left(mean_{1}-mean_{2}\right)}^{2}}$, *n*_1_ = *k*×*n*_2_, was used for the calculation. Z_1−**α**/2_ = 1.96, Z_1−**β**_= 0.84, according to the pre-experimental results, the mean ± standard deviation ($\overset{-}{\chi }$ ± SD) of ePRO group is 50 ± 10, and the ($\overset{-}{\chi }$ ± SD) of the control group is 43 ± 10. Taking into account the dropouts and lost follow-up rate of about 20%, we will finally enroll 50 participants in each group.

### Randomization and Allocation

Following the signing of a consent form, participants are allocated in a 1:1 ratio to either ePROs-based group or control group based on the computer-generated random. Group numbers were sealed into the non-transparent envelopes kept by a specially appointed person. Because of the nature of interventions, the blinding of participants and investigators is impossible. But the data collectors who help to administer ePROs collection will be blinded to group allocation.

### Intervention and Control

The primary early advantage of the ePROs applet development process was the product development based on the human-centered design (HCD) philosophy and two-drill design model. Stakeholders including children, parents, researchers in related fields, clinical medical staff, software engineers, and so on were included. Sufficient demand mining was carried out through qualitative research methods to ensure the scientific nature of information system product design in the stage of “discovery” and “definition.” In the whole process of “development,” the agile software development method is adopted, that is, the gradual development of modules and functions is completed, and the results of the development team are tested after the initial completion of each module development, and the vulnerabilities are corrected immediately, so that the software is always in the state of trial and operation in the development process. Under the continuous iteration of module development and testing, overall development and testing, the final development of the product was completed. ePROs give full consideration to the children's users these specific population characteristics, such as cartoon interface, voice assistant function, games, incentive function, and so on.; at the same time, the content, and function of development are the combination of theoretical research, literature research, expert consultation, and so on, so the final small program overall harvest in the usability test users more positive evaluation.

After enrollment, investigators help the patients develop a WeChat mini-program (ePROs) by scanning the code, and the participants are instructed on how to use the ePRO. They are required to complete the first ePROs questionnaires (including demographic questionnaire, PedsQL^TM^3.0-RM, and C-Ped-PROMIS) during hospitalization (baseline, typically within 48 h after admission). The patient disease-associated questionnaire was filled out by the investigator before the patients were discharged from hospital. The follow-up period is 12 months, and we will collect the ePROs questionnaires at four time points (1, 3, 6, and 12 months after discharge). Then, 1 week before these four scheduled time points, a WeChat reminder message was sent to patients automatically to remind them to complete the PedsQL^TM^3.0-RM and C-Ped-PROMIS. If they do not respond within the scheduled time, we will send a reminder message again. If they still do not respond within 2 days after reminding, a telephone follow-up within 24 h will be conducted. Patients who did not answer after three phone calls within 24 h were considered “lost to follow-up,” and they were censored at the last follow-up date.

ePROs-based group will receive symptom management, the specific process as follows: when there are one or more one C-Ped-PROMIS items exceed the preset intervention threshold (T-scores ≥50), the “early warning symptom” will be triggered. The investigators will receive real-time warning information on WeChat, and they will generate some interventions based on the reported scores and send it to the patient through the “symptom intervention” module. Interventions are developed by a multidisciplinary team (including rheumatologists, specialist nurses, therapists, and others) based on the clinical experience and updated clinical practice guidelines. The control group will only fill in the ePROs questionnaires and do not trigger the “early warning symptom.” During hospitalization, the doctors and nurses manage symptoms based on their judgment rather than the scores of C-Ped-PROMIS. After discharge, they will imply the standard symptom interventions according to the patients' disease condition and treatment effects. Patients are suggested to contact the clinical team immediately in case of serious impairments.

### Outcomes and Measurement

#### Primary Outcome

The primary outcome of this study is the mean T-scores of patients in the ePROs-based group and the control group. The C-Ped-PROMIS is the primary ePRO tool, and it has eight short forms for assessing the child's symptoms and quality of life during the previous 7 days. Each form has a single dimension, and the 5-level Likert scoring method scale with the answers “never” (0) and “almost” always (4) is used for each item. Raw scores of each form are converted to standardized T-scores with a mean of 50 and a standard deviation of 10. Only all forms are answered completely and performed this conversion.

#### Secondary Outcomes

The second outcomes of our study are the trajectories of C-Ped-PROMIS and HRQOL. We assessed the longitudinal changing trend of the mean T-scores of the C-Ped-PROMIS and the mean score of the PedsQL^TM^3.0-RM from baseline to 12 months after discharge. Furthermore, we examined the relationships between changes in C-Ped-PROMIS and changes in the HRQOL by evaluating the T-scores changes and PedsQL^TM^3.0-RM score changes among patients in the ePROs-based group and those in the control group.

The PedsQL^TM^3.0-RM (33) is a mature and specific tool for measuring the HRQOL of children with rheumatic diseases. It includes an age scale with five dimensions: pain (four items), daily activities (five items), treatment (seven items), worry (three items), and communication (three items), 22 items in total. There are five response options: “never,” “almost never,” “sometimes,” “almost always,” “always,” to which the scores 100, 75, 50, 25, and 0 are given, respectively. The scores of each dimension and total scale are the total score of each item divided by the number of response items, the score ranges from 0 to100, with higher scores indicating better HRQOL. The Chinese version of PedsQL^TM^3.0-RM has been verified to be a good instrument with acceptable reliability and validity and is suitable for assessing the quality of life among Chinese children with RA (37).

#### Other Data

The patient's disease-associated will be assessed through a separate disease-associated assessment questionnaire. Disease activity will be assessed by the JADAS, which includes the four measures and is feasible and possesses content validity. The presence or absence of complications, biochemical markers (erythrocyte sedimentation rate (ESR), C-reactive protein (CRP), tumor necrosis factor, and IL-1β), and laboratory tests (joint magnetic resonance imaging (MRI) and B-mode ultrasonography) are all recorded. During the initial assessment, a demographic questionnaire is sent to patients which included age, sex, race, ethnicity, and economic status. In addition, the completion time of the ePROs system, intervention compliance, and loss to follow-up will also be recorded.

### Statistical Analyses

SPSS 20.0 will be used for statistical analysis. Intention-to-treat analyses will be conducted by including all available data in the analysis. Missing data will be imputed by the multiple imputation method. A two-sided *p* < 0.05 is considered to be statistically significant. Data are first subjected to the normality tests, continuous variables that conformed to the normal distribution will be presented as the mean (SD), and do not conform to a normal distribution will be expressed as median (IQR). Categorical variables will be presented as frequencies or proportions. Comparisons between groups will be conducted using the Wilcoxon and Fisher's exact tests. Trajectories of PROs will be compared in two groups by repeated measures ANCOVA, as implemented under the general linear model. The MPLUS will be used to sort and analyze the data, and the LTA model was used to evaluate the intervention effect. In addition, subgroup analysis was carried out according to subgroups of JIA.

### Data Quality and Management

(1) Establishing a trusting relationship: participants are all recruited by clinical frontline medical staff in the rheumatology department, and they have established a well-with patients and their families. The researchers explain in detail the purposes, procedures, methods, and significance of the research to the object, and sign an informed consent form. (2) Controlling bias: select senior investigators with highly responsible and strong rheumatology knowledge and investigation in the name of the hospital for reducing loss to follow-up and withdrawal. In data analysis, for the missing data, the imputation method is used to replace it. (3) Avoiding contamination and interference: the subjects in the experimental group and the control group will be physically isolated as far as possible until the entire longitudinal study. In addition, investigators will have an adequately communicates with patients to ensure the validity of the filling.

## Discussion

To our knowledge, this trail is the first RCT study in the field of Pediatric PROMIS investigating the effect of symptom management based on ePROs. Few studies have applied the Pediatric PROMIS to monitoring symptoms in patients with JIA. All or some domains of Pediatric PROMIS are used as one of the outcome indicators of drug clinical trials. In an observational, prospective, Childhood Arthritis and Rheumatology Research Alliance (CARRA) Registry study, PROMIS pain interference and mobility are assessed as the secondary main outcomes (24). The precision and discriminatory abilities of Pediatric PROMIS have been rigorously studied by Brandon, and the results indicated that the health domain and the subject of the report (self-report or parent proxy-report) are the main influencing factors (23). Different from these researches, this study adds a new function of “symptom intervention” module to the ePRO and verifies its effectiveness. JIA has a major impact on patient's daily life, in addition to actively treating patients with JIA, continuous symptom monitoring and implementation of intervention measures after discharge are important for the improvement of disease in patients with JIA, which is a long-term process (38). In the past, most doctors and nurses were only concerned about symptom management during hospitalization, management after discharge is insufficient, and the lack of suitable remote management tools is one of the important reasons. In this trial, we implement remote, dynamic, and individualized interventions for patients based on ePROs through the symptom management module.

Our study will contribute to establishing evidence for the symptom management of JIA based on ePROs. The possible implications of shreds of evidence include the following: (1) determining whether ePRO-based symptom management is better than routine management, (2) identifying whether ePRO-based symptom management is feasible and acceptable in clinical practice, (3) identifying whether symptom management based on ePROs can improve HRQOL in the patients with JIA, (4) providing a reference for the long-term symptom management of various diseases in pediatrics in the future.

Further, the strengths of this planned study include that this is a higher-level evidence research design. We will follow patients for a year and collect the data at five time points. The contents filled in the ePROs can be directly exported, avoiding the error of the second data entry. There are also many limitations in this trial. First, this trial is a single-center research, so the sample recruited in this study may confer some selection bias. Second, the follow-up time is longer, which may result in more patients lost to follow-up. Regarding the bias of loss to follow-up, although we will control the loss to follow-up by fully explaining the research process when recruiting, establishing a good nurse–patient relationship during hospitalization, and recording more than two contact details, for the 1-year study, we still have great concerns about the response rate of patients. Third, due to the natural nature of research, intervention will be blinded to participants and investigators, which may increase the measurement bias. Fourth, taking into account ethical constraints, when the T-scores of the symptom domain reported by the control group exceed the preset upper threshold (or lower threshold), we will inform the patients, which may weaken the effect of management to a certain extent.

In conclusion, this study will assess the effect of symptom management for patients with JIA based on ePROs and provide data on the feasibility of remote, dynamic, individualized monitoring and intervention of patients with JIA.

## Ethics Statement

The studies involving human participants were reviewed and approved by Ethics Committee of Shanghai Children's Medical Center, Shanghai Jiao Tong University School of Medicine (SCMCIRB-YN2021001). Written informed consent to participate in this study was provided by the participants' legal guardian/next of kin.

## Author Contributions

BS, SS, and HCu conceptualized and designed the study, drafted the initial manuscript, and reviewed and revised the manuscript. YL, HCh, HJ, and JX acquired the data, drafted the initial manuscript, and reviewed and revised the manuscript. ZL and YJ conceptualized and designed the study, revised the article critically, and approved the version to be published. All authors approved the final manuscript as submitted and agreed to be accountable for all aspects of the work.

## Funding

This work was supported by Project of Shanghai Municipal Health Commission (under grant number 201740233), National Natural Science Foundation of Shanghai Children's Medical Center (under the grant number YJG-SCMC2021-13), and Innovative research team of high-level local universities in Shanghai. Shanghai Jiao Tong University School of Medicine: Nursing Development Program.

## Conflict of Interest

The authors declare that the research was conducted in the absence of any commercial or financial relationships that could be construed as a potential conflict of interest.

## Publisher's Note

All claims expressed in this article are solely those of the authors and do not necessarily represent those of their affiliated organizations, or those of the publisher, the editors and the reviewers. Any product that may be evaluated in this article, or claim that may be made by its manufacturer, is not guaranteed or endorsed by the publisher.
